# Mie scatterers bring a resonator to an exceptional point

**DOI:** 10.1038/s41377-023-01258-4

**Published:** 2023-09-04

**Authors:** Kai Hong, Lin Chen

**Affiliations:** Wuhan National Laboratory for Optoelectronics and School of Optical and Electronic Information, Huazhong University of Science and Technology, Wuhan, 430074 China

**Keywords:** Optics and photonics, Optical physics

## Abstract

Exceptional points have given rise to many intriguing optical phenomena that are of fundamental importance for a variety of breakthrough technologies. The pre-defined Mie scatterers can bring a resonator to an exceptional point, and result in enhanced quality factor through coherently suppressing the backward scattering.

Exceptional points (EPs) are degenerate points where two or more eigenvalues and eigenvectors coincide simultaneously in non-Hermitian systems^1–3^. Optical platforms provide a powerful tool for investigating the EP-related physics, such as coupled waveguides and microcavities. EPs have led to many exotic phenomena such as loss-induced suppression^4^, directional and mode-selective lasing^5–7^, enhanced sensing sensitivity^8,9^, and chiral switching^10–12^. There are several common methods to bring an optical system to an exceptional point. One approach involves tuning the coupling strength between the coupled components with balanced optical gain and loss, as well as adjusting the loss imbalance with a fixed coupling strength. Another method involves controlling the coupling between the modes of a physical system.

In the paper entitled “Chiral exceptional point and coherent suppression of backscattering in silicon microring with low-loss Mie scatterer” published in eLight^13^, Prof. Sahin K. Özdemir’s group and Prof. Tingyi Gu’s group proposed a new approach to bring an optical resonator using low-loss Mie scatterers. They precisely control the location, size, and geometry of the Mie scatterers in silicon microrings, thereby manipulating the amplitude, phase, and direction of transmission and reflection. Their theoretical analysis shows that two embedded Mie scatterers, one symmetric and one asymmetric, or two non-identical asymmetric Mie scatterers, provide sufficient flexibility to tune the system toward or away from an EP. Moreover, the Mie scatterers effectively suppress the backward scattering caused by fabrication-induced Rayleigh scattering. A silicon microring without Mie scatterers supports both clockwise (CW) and counterclockwise (CCW) modes. The coupling and frequency detuning between the CW and CCW modes result in a reduced optical quality factor due to backward scattering^14^ (Fig. 1a). However, by introducing Mie scatterers to the silicon microring, it can operate at an EP, exhibiting mode degeneracy and complete suppression of backward scattering. The Mie scatterers enhance the quality factor measured on the transmission port by coherently suppressing the backscattering from the waveguide surface roughness (Fig. 1b).

**Fig. 1:**
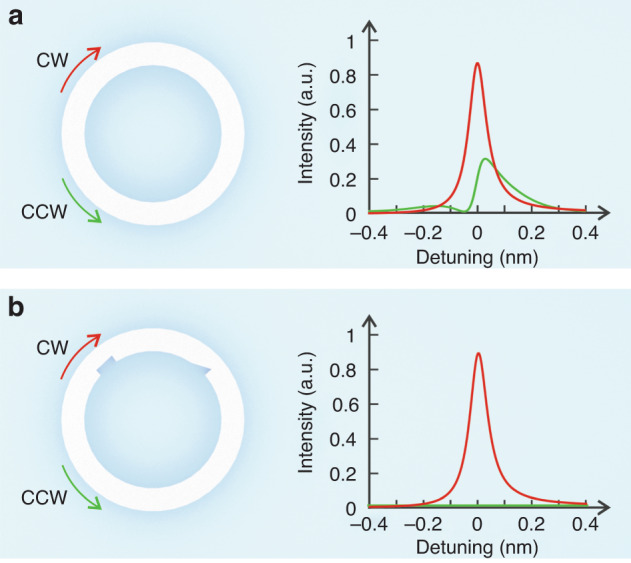
Silicon microring. **a** A silicon microring without Mie scatterers supports both CW and CCW modes. The CW mode is comparable to CCW modes. **b** A silicon microring can support the CW mode only by controlling the Mie scatterers to bring the system to an EP. The CCW mode is highly suppressed and an infinitesimal, as opposed to the CW mode

The researchers employed a Smith Chart-based “optical impedance matching” approach to design a silicon microring with Mie scatterers operating at an EP. To experimentally demonstrate the high-quality factor and suppression of backward scattering in the designed silicon microring, they fabricated a series of silicon microrings operating at or near the EP, all having the same surface roughness levels. The experimental results are in line with the expected outcomes. The measured quality factor Q increased from 16,300 (for the silicon microring without Mie scatterers) to 21,300 (for the silicon microring with Mie scatterers operating at an EP). In addition, it was observed that the silicon microring operating at an EP exhibited minimal back-reflection. Furthermore, the experimental results showed that the system is not sensitive to dispersion and is robust to structural parameters.

In contrast to whispering gallery mode resonators^6^, the silicon microring with low-loss Mie scatterers replaces fiber tapered coupling with on-chip silicon waveguides with gratings. The completely on-chip controlled structures offer the advantage of higher integration density. Compared to the microgear photonic crystal ring^15^, the silicon microring with Mie scatterers can coherently suppress backscattering from the waveguide surface roughness and provides improved stability. The inclusion of Mie scatterers in the silicon microring not only opens up new avenues for studying chiral silicon photonics but also contributes to advancing the understanding of EPs and non-Hermitian physics.
